# Association of Remote Monitoring With Survival in Heart Failure Patients Undergoing Cardiac Resynchronization Therapy: Retrospective Observational Study

**DOI:** 10.2196/14142

**Published:** 2019-07-26

**Authors:** Peter Bogyi, Mate Vamos, Zsolt Bari, Balazs Polgar, Balazs Muk, Noemi Nyolczas, Robert Gabor Kiss, Gabor Zoltan Duray

**Affiliations:** 1 Department of Cardiology Hungarian Defence Forces Medical Centre Budapest Hungary; 2 Basic and Translational Medicine Karoly Racz School of PhD Studies Semmelweis University Budapest Hungary; 3 Department of Cardiology University Hospital Frankfurt Goethe University Frankfurt Germany

**Keywords:** survival, CRT-D, remote monitoring, telemedicine, heart failure

## Abstract

**Background:**

Remote monitoring is an established, guideline-recommended technology with unequivocal clinical benefits; however, its ability to improve survival is contradictory.

**Objective:**

The aim of our study was to investigate the effects of remote monitoring on mortality in an optimally treated heart failure patient population undergoing cardiac resynchronization defibrillator therapy (CRT-D) implantation in a large-volume tertiary referral center.

**Methods:**

The population of this single-center, retrospective, observational study included 231 consecutive patients receiving CRT-D devices in the Medical Centre of the Hungarian Defence Forces (Budapest, Hungary) from January 2011 to June 2016. Clinical outcomes were compared between patients on remote monitoring and conventional follow-up.

**Results:**

The mean follow-up time was 28.4 (SD 18.1) months. Patients on remote monitoring were more likely to have atrial fibrillation, received heart failure management at our dedicated heart failure outpatient clinic more often, and have a slightly lower functional capacity. Crude all-cause mortality of remote-monitored patients was significantly lower compared with patients followed conventionally (hazard ratio [HR] 0.368, 95% CI 0.186-0.727, *P*=.004). The survival benefit remained statistically significant after adjustment for important baseline parameters (adjusted HR 0.361, 95% CI 0.181-0.722, *P*=.004).

**Conclusions:**

In this single-center, retrospective study of optimally treated heart failure patients undergoing CRT-D implantation, the use of remote monitoring systems was associated with a significantly better survival rate.

## Introduction

Remote monitoring of cardiac implantable electronic devices has proved to be beneficial on several clinical endpoints. The first studies confirmed the feasibility of early, automatic detection of technical issues [1], and recognition of a new onset of atrial fibrillation [2]. Randomized studies proved that remote monitoring could reduce time to evaluate arrhythmic events [3], decrease mean length of cardiovascular hospitalizations [4], and could significantly lower the number of appropriate or inappropriate shocks [5]. This method is also able to reduce in-office implantable cardioverter defibrillator (ICD) follow-up burden safely [6] Remote monitoring also provided early detection of heart failure events and reduced the number of urgent in-office visits and total health care use in patients with ICD or cardiac resynchronization systems in diverse clinical studies [7-9].

Moreover, registry data suggest a potential survival benefit in patients on remote monitoring [10,11]. The most important limitation of these register-based reports is the lack of randomization and the paucity of clinical characteristics that would make a more accurate comparison possible. In the multicenter EFFECT study, remote monitoring was associated with reduced deaths and cardiovascular hospitalizations in patients with ICD. [12] In the randomized, controlled, international multicenter IN-TIME study, a significant survival benefit of implant-based multiparameter telemonitoring was demonstrated over the standard of care in patients with heart failure and implanted dual-chamber ICD or cardiac resynchronization therapy defibrillator (CRT-D) [13].

However, a recent randomized trial on remote monitoring (MORE-CARE) could not reduce mortality or risk of cardiovascular or device-related hospitalizations [14]. Furthermore, the REM-HF multicenter randomized study showed similar outcomes among patients with heart failure and cardiac implantable electronic devices utilizing remote monitoring with weekly downloads and a prespecified follow-up approach [15].

Concerning these contradictory results, we aimed to investigate the effects of remote monitoring on mortality in an optimally treated heart failure patient population undergoing CRT-D implantation in a large-volume tertiary referral center.

## Methods

### Study Patients

The population of this single-center, retrospective, observational study included consecutive patients receiving CRT-D devices in the Medical Centre of the Hungarian Defence Forces (Budapest, Hungary) from January 2011 to June 2016. Indication for implantation was established according to current guideline recommendations of the European Society of Cardiology [16,17]. CRT-ICDs from various manufacturers were used (Biotronik, Germany; ELA/Sorin, Italy; Guidant/Boston Scientific, Marlborough, MA; Medtronic, Minneapolis, MN; and St Jude Medical/Abbott, St Paul, MN). Choice of device type was left to the implanting physician’s discretion.

### Study Design

The possibility of remote monitoring was offered to every patient implanted with a wireless telemetry-capable CRT-D device. Patients who consented to remote monitoring formed the remote monitoring group. During the inclusion period, remote monitoring systems of two manufacturers (Medtronic CareLink Network, Medtronic Inc, Minneapolis, MN; and Home Monitoring Service Center, Biotronik GmbH & Co KG, Germany) were available in our center. The control group consisted of CRT-D recipients, who were followed up in our outpatient device clinic without remote monitoring.

The CareLink network operates with scheduled transmissions defined by the physician, and unscheduled transmissions, which can be triggered both by the patient (manual transmission) and by the device itself (alert event). Scheduled remote transmissions were set up every 3 months [18]. It was also recommended to patients to send manual transmissions in case of palpitation, syncope, or worsening of heart failure symptoms. Alert programming was set up according to previously published Medtronic-sponsored trials as follows: OptiVol alert (nominal fluid index ≥60 Ω-day), daily atrial fibrillation burden greater than 6 hours per day, ventricular rate during atrial fibrillation greater than 100 bpm for 6 hours, two or more shocks delivered, all therapies exhausted, lead or device integrity alert, lead impedance out of range, recommended replacement time, and end of service [7,18,19].

Home Monitoring uses a mobile phone network to transmit device data automatically on a daily basis, as well as instantly on the occurrence of a potentially clinically relevant event [20]. These parameters include device and battery status, pacing impedances, bradycardia, tachycardia, and CRT statistics, mean heart rates, patient activity, heart rate variability, and current programming of the device [21].

In-office visits were recommended to patients on remote monitoring without symptoms at least once a year [22]. Remote transmissions were evaluated every day by a team consisting of cardiology trainees and consultant electrophysiologists. In the case of suspected heart failure progression, a heart failure specialist was involved additionally. Transmissions were labeled as clinically nonsignificant, clinically relevant (yellow alert), or highly urgent (red alert). If a clinically relevant event was perceived, patients were contacted via phone calls and were invited to the clinic for a personal visit in a week. Definitions of a clinically relevant event for the two remote monitoring systems are summarized in Multimedia Appendix 1. Transmissions with highly urgent content defined as ventricular arrhythmias treated with more than one ICD shock or system integrity alert were handled within 24 hours. Patients with missed transmissions longer than 4 weeks were also contacted. Follow-up of patients in the control group was performed at intervals of 3 to 6 months.

Patient demographics, comorbidities, pharmacotherapy, electrocardiogram characteristics, echocardiography, and laboratory data were collected at enrollment and during scheduled remote checks or in-office visits.

All patients on remote monitoring signed a related informed consent form. The study complied with the Declaration of Helsinki, and the study protocol was approved by the Institutional Ethics Committee of Hungarian Defence Forces Medical Centre, Budapest, Hungary.

### Study Endpoints

The primary objective of this study was to compare the mortality of remote-monitored patients with patients on conventional follow-up. Survival was assessed as the time from CRT-D implantation to all-cause mortality. Mortality data were retrieved using the Hungarian National Health Fund Death Registry. The unique health insurance number of a patient is deactivated immediately after death. The secondary endpoint was the response to resynchronization therapy at the visit at 6 to 12 months, defined as 5% absolute increase in left ventricular ejection fraction. The number of all ambulant visits, device clinic visits, and heart failure outpatient clinic visits were also analyzed and compared between the two patient groups.

### Statistical Analysis

Statistical analysis was performed using PASW Statistics software, version 18.0.0 (WinWrap Basic, Polar Engineering and Consulting). The Kolmogorov-Smirnov test was used to evaluate the normal distribution of continuous data. The chi-square test was applied to test for categorical variables; the two-sample *t* test or the Mann-Whitney *U* test was used for continuous variables among patient groups.

To assess the effects of remote monitoring on survival, the Cox proportional hazards regression model was used. Univariate analysis was performed for the following variables: age; gender; heart failure management; upgrade procedure; secondary prevention; ischemic etiology; atrial fibrillation; hypertension; hyperlipidemia; diabetes mellitus; stroke; peripheral artery disease; chronic obstructive pulmonary disease; New York Heart Association (NYHA) functional class; left ventricular ejection fraction; QRS duration; left bundle branch block; estimated glomerular filtration rate; hemoglobin; and therapy with platelet aggregation inhibitors, beta blockers, angiotensin-converting-enzyme inhibitors or angiotensin-receptor blockers, mineralocorticoid antagonists, diuretics, digitalis, amiodarone, and statin. All variables with *P* ≤.10 on univariate analysis were included in the multivariate Cox models. Two-sided *P* values <.05 were considered statistically significant. Survival curves were constructed according to the Kaplan-Meier method and compared with the Cox proportional hazard model. To check for interaction between survival and the specific remote monitoring system, all-cause mortality was also compared between the subgroups of patients on CareLink and on Home Monitoring systems.

## Results

**Figure 1:**
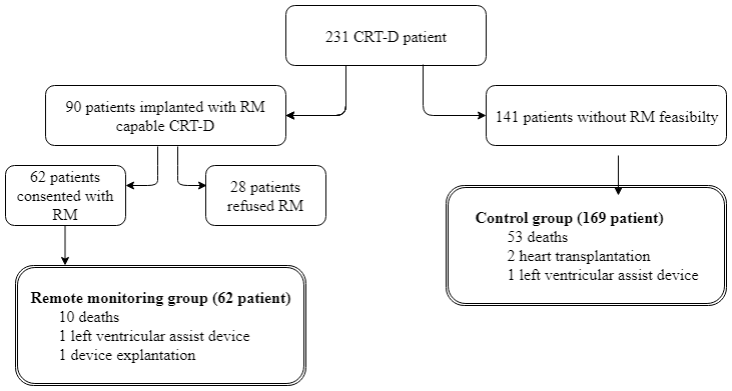
Study flow diagram. CRT-D: cardiac resynchronization therapy defibrillator; RM: remote monitoring.

A total of 231 CRT-D recipients were included in this study. Of the 90 patients implanted with remote monitoring-capable devices, 62 consented to receive a remote monitor (41 of 56 patients with Medtronic and 21 of 34 with Biotronik devices; Figure 1).

Detailed patient baseline data are summarized in Table 1. Despite the nonrandomized nature of the study, there were only a few significant differences between the two patient groups: patients on remote monitoring were more likely to have atrial fibrillation and have received heart failure management more often at our dedicated heart failure outpatient clinic. They also had a slightly lower NYHA functional class.

**Table 1:** Patient baseline characteristics (N=231).

Characteristic		Remote monitoring group (n=62)	Control group (n=169)	*P* value
Age (years), mean (SD)		64.0 (9.9)	65.6 (10.8)	.26
Male, n (%)		51 (82)	133 (78.7)	.55
Left ventricular ejection fraction (%), mean (SD)		27.3 (6.5)	26.3 (5.8)	.29
NYHA^a^ functional class, mean (SD)		2.4 (0.7)	2.6 (0.7)	.05
QRS duration (ms), mean (SD)		147 (28)	154 (29)	.08
Left bundle branch block, n (%)		46 (74)	130 (76.9)	.71
Upgrade procedure, n (%)		10 (16)	46 (27.2)	.08
Primer prevention, n (%)		51 (82)	136 (80.5)	.76
Heart failure outpatient clinic management, n (%)		39 (63)	77 (45.6)	.02
Ischemic etiology, n (%)		33 (53)	95 (56.2)	.68
Hypertension, n (%)		46 (74)	131 (77.5)	.14
Diabetes, n (%)		25 (40)	54 (31.9)	.23
Hyperlipidemia, n (%)		25 (40)	51 (30.2)	.14
Atrial fibrillation, n (%)		25 (40)	44 (26.0)	.03
Chronic obstructive pulmonary disease, n (%)		9 (15)	22 (13)	.76
Estimated glomerular filtration rate (mL/min/1.73 m^2^), mean (SD)		55.9 (17.6)	58.3 (20.3)	.58
Hemoglobin (g/L), mean (SD)		133 (15)	133 (16)	.95
**Concomitant medications, n (%)**				
	Beta blocker	62 (100)	166 (98.2)	.29
	ACEi^b^/ARB^c^	61 (98)	167 (98.8)	.89
	Mineralocorticoid receptor antagonist	58 (94)	158 (93.5)	.98
	Diuretic	56 (90)	157 (92.9)	.51
	Amiodarone	14 (23)	51 (30.2)	.25
	Digoxin	16 (26)	26 (15.4)	.06

^a^NYHA: New York Heart Failure Association.

^b^ACEi: angiotensin-converting-enzyme inhibitor.

^c^ARB: angiotensin-receptor blocker.

**Figure 2:**
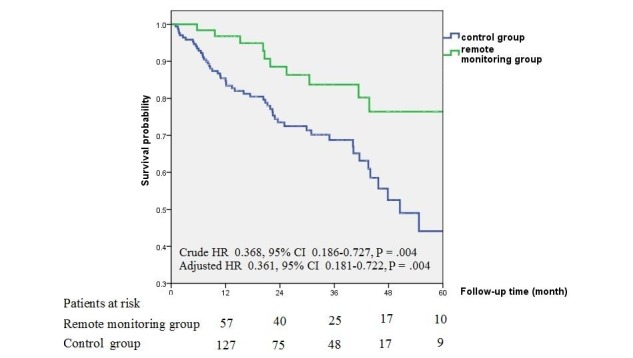
Kaplan-Meier curves for all-cause mortality by follow-up type (remote monitoring vs conventional follow-up). HR: hazard ratio.

During the average follow-up time of 28.4 (SD 18.1) months, 63 patients died, 2 underwent heart transplantation, 2 received a left ventricular assist device, and in 1 case device explantation was performed due to infection. Crude all-cause mortality of remote-monitored patients was significantly lower compared with patients followed conventionally (hazard ratio [HR] 0.368, 95% CI 0.186-0.727, *P*=.004; Table 2; Figure 2). The survival benefit remained statistically significant after adjustment for important baseline parameters (adjusted HR 0.361, 95% CI 0.181-0.722, *P*=.004; Table 3; Figure 2). The survival benefit did not differ between the remote monitoring systems (ie, CareLink vs Home Monitoring; *P*=.79).

Echocardiographic response to cardiac resynchronization therapy at 6 to 12 months was more often observed in patients on remote monitoring (41.9%, 26/62 vs 31.9%, 54/169); however, this difference was statistically nonsignificant (Table 4).

**Table 2:** Predictors of mortality (univariate Cox regression).

Predictor	Hazard ratio (95% CI)	*P* value
Age	1.049 (1.020-1.079)	.001
Male gender	1.996 (0.949-4.195)	.07
Remote monitoring	0.368 (0.186-0.727)	.004
Heart failure management	0.584 (0.351-0.970)	.04
Upgrade	1.869 (1.112-3.140)	.02
Secondary prevention	1.488 (0.820-2.698)	.19
Ischemic etiology	1.373 (0.285-2.284)	.22
Atrial fibrillation	1.799 (1.083-2.990)	.02
Hypertension	2.226 (1.128-4.395)	.02
Hyperlipidemia	0.811 (0.469-1.402)	.45
Diabetes mellitus	1.092 (0.650-1.836)	.74
Stroke	1.699 (0.861-3.354)	.13
Peripheral artery disease	1.155 (0.663-3.668)	.31
Chronic obstructive pulmonary disease	1.239 (0.587-2.616)	.57
Platelet aggregation inhibitor	1.180 (0.714-1.948)	.52
Beta blocker	0.407 (0.099-1.684)	.22
ACEi^a^/ARB^b^	0.692 (0.096-5.007)	.72
Mineralocorticoid antagonist	0.769 (0.349-1.696)	.52
Diuretics	1.606 (0.503-5.217)	.42
Digitalis	0.772 (0.410-1.454)	.42
Amiodaron	1.986 (1.192-3.309)	.008
Statin	1.243 (0.725-2.130)	.43
NYHA^c^ functional class	1.394 (1.017-1.910)	.04
Left ventricular ejection fraction	1.028 (0.987-1.071)	.18
QRS duration	1.001 (0.992-1.010)	.88
Left bundle branch block	1.308 (0.961-1.782)	.09
Estimated glomerular filtration rate	0.991 (0.978-1.005)	.23
Hemoglobin	1.002 (0.998-1.007)	.26

^a^ACEi: angiotensin-converting-enzyme inhibitor.

^b^ARB: angiotensin-receptor blocker.

^c^NYHA: New York Heart Failure Association.

**Table 3:** Independent predictors of mortality (multivariate Cox regression).^a^

Predictor	Hazard ratio (95% CI)	*P* value
Age	1.035 (1.007-1.065)	.02
Remote monitoring	0.361 (0.181-0.722)	.004
Amiodaron	1.732 (1.032-2.907)	.04
Atrial fibrillation	1.727 (1.019-2.926)	.04

^a^Cases with missing values 0%.

**Table 4:** Echocardiographic response to cardiac resynchronization therapy at 6 to 12 months (N=231).

Left ventricular ejection fraction (%)	Remote monitoring group (n=62)	Control group (n=169)	*P* value
Baseline, mean (SD)	27.3 (6.5)	26.3 (5.8)	.29
Follow-up, mean (SD)	33.2 (8.2)	32.3 (9.2)	.34
Change, mean (SD)	6.9 (8.0)	6.2 (8.2)	.48
Absolute improvement ≥5%, n (%)	26 (41.9)	54 (31.9)	.57

The total number of follow-up controls tended to be higher in the remote monitoring group compared with patients undergoing conventional follow-up (4.6 visits per patient per year vs 3.9 visits per patient per year, *P*=.08). This was because patients on remote monitoring presented at our specialized heart failure outpatient clinic more often (1.9 visits per patient per year vs 1.1 visits per patient per year, *P*=.003; Table 5).

Of the 41 patients followed with the CareLink system, 1083 transmissions were received during the follow-up period. Seven transmissions contained highly urgent clinical information (four appropriate shock episodes and three lead integrity alerts). Moreover, two patients reached an elective replacement indicator. In addition, 396 transmissions included OptiVol alerts. Telemonitoring observations in the 21 patients on the Biotronik Home Monitoring system were as follows: 3 red alerts (two electric storms, one end of service) and 85 yellow alerts (8 sustained ventricular arrhythmias requiring antitachycardia pacing or shock therapy, 36 supraventricular tachyarrhythmia, 36 low biventricular pacing percentage, and 5 elevated left ventricular threshold).

**Table 5:** Ambulatory visits during follow-up (N=231).

Visit type		Remote monitoring group (n=62)	Control group (n=169)	*P* value
**All ambulant visits**				.08
	Visits, n	711	1187	
	Visits per patient per year, mean (SD)	4.6 (3.0)	3.9 (3.0)	
**Device clinic visits**				.95
	Visits, n	435	889	
	Visits per patient per year, mean (SD)	3.1 (2.4)	2.9 (2.5)	
**Heart failure outpatient clinic visits**				.003
	Visits, n	347	344	
	Visits per patient per year, mean (SD)	1.9 (2.4)	1.1 (2.0)	

## Discussion

### Principal Findings

In this single-center, retrospective, observational study of 231 CRT-D recipients, use of remote monitoring was associated with better survival compared with patients undergoing conventional follow-up. The total number of follow-up visits was not reduced with this technique in our cohort.

### Comparison With Prior Work

Remote monitoring systems proved to be feasible, reliable, accessible, and were supported by the current guideline recommendations [16,23]. They are still underutilized despite the clear advantages, such as early identification of device malfunction or arrhythmic events. Regarding survival benefit, the available clinical data are controversial [10,13,24-27].

There are several proposed mechanisms contributing to the improved clinical outcome: early detection of clinically relevant arrhythmias (ie, atrial fibrillation or ventricular tachycardia) and early recognition of device malfunctions or suboptimal programming, which avoids unnecessary shocks and achieves proper percentage of biventricular pacing, respectively.

Moreover, the number of OptiVol alerts and the related visits at the heart failure outpatient clinic suggest that the observed mortality benefit associated with remote monitoring was also driven by early response to cardiac decompensation in our study. OptiVol is a detection algorithm developed for early recognition of cardiac decompensation using changes of intrathoracic impedance as a marker of lung fluid status [28]. This method, used for the detection of cardiac decompensation, is considered to be a very sensitive but less specific tool, which leads to a high number of false positive alerts. We have previously described a refined device diagnostic algorithm based on parameters of low activity level, high nocturnal heart rate, and suboptimal biventricular pacing, which could significantly improve clinical reliability of OptiVol alerts [9]. However, a recent OptiLink heart failure study analyzing this technology failed to demonstrate any difference in the composite endpoint of all-cause mortality and cardiovascular hospitalization [29]. Notably, only 30.3% of intrathoracic fluid index threshold crossing led to medical action, and it led to altered medication in only 26.0% of patients.

Our analysis shows a survival benefit for patients on remote monitoring; therefore, we are convinced that remote monitoring should be part of the follow-up of patients with CRT-D devices. One possible reason for the conflicting results of previous studies is the wide variety of actions on remote monitoring findings. A recently published meta-analysis by Stockburger et al [30] showed that device-based remote monitoring strategies specifying close-meshed comprehensive data acquisition and defined treatment interventions are able to significantly reduce total mortality and cardiovascular mortality, whereas remote data acquisition alone without specified treatment interventions appears to be ineffective on hard endpoints. The significantly increased number of visits in the heart failure clinic in our study supports this hypothesis, as the demonstrated survival benefit could have been achieved by more frequent follow-up and focused treatment on the high-risk patients.

Additional clinical factors can also modify the clinical benefit of remote monitoring, such as the time from implantation to initiation of remote monitoring [31], the adherence to this technique [11], or the frequency of data transmission. Two recently published meta-analyses demonstrated that significant mortality benefit was only seen in the subset of trials using a daily transmission verification (ie, Biotronik Home Monitoring System) [26,27]. A possible explanation for this difference is that the rate of events recognized within 24 hours is the highest among manufacturers with the Biotronik Home Monitoring system [32]. However, the type of remote monitoring system did not influence the survival benefit in our patient cohort.

### Limitations

This research is a single-center, retrospective study with all the consequential limitations. Despite the adjustment for the most relevant baseline cofounders, a residual selection bias can not be completely excluded. Moreover, the choice of device was not randomized but was left to the implanting physician, which may have modified our results. The patient’s decision to consent to remote monitoring may also have modified the results. The potential sources of bias should also be addressed. The most important one is the patient’s decision to consent with a remote monitoring program. Patients with better adherence and motivation are more likely to participate in such programs, which might have improved the outcomes of patients in the remote monitoring group.

### Conclusion

In this single-center, retrospective study of optimally treated heart failure patients undergoing CRT-D implantation, the use of remote monitoring systems was associated with significantly better survival. However, a higher number of follow-up visits in the heart failure outpatient clinic was needed, which suggests that this survival benefit could be achieved by an increased and focused effort to follow and treat high-risk patients. Our results call for further randomized studies with a standardized action plan after certain telemonitoring observations to define the optimal role of this technology in the follow-up of heart failure patients.

## Appendix

Multimedia Appendix 1 Definitions of clinically relevant events.

## Abbreviations

ACEi: angiotensin-converting-enzyme inhibitor
ARB: angiotensin-receptor blocker
CRT-D: cardiac resynchronization therapy defibrillator
HR: hazard ratio
ICD: implantable cardioverter defibrillator
NYHA: New York Heart Association
