# Supplementary material for: Association of Remote Monitoring With Survival in Heart Failure Patients Undergoing Cardiac Resynchronization Therapy: Retrospective Observational Study
Source: J Med Internet Res. 2019 Jul 26;21(7):e14142. doi: 10.2196/14142 (PMC6688436; doi:10.2196/14142)
Supplement: Multimedia Appendix 1 [file jmir_v21i7e14142_app1.pdf]

Event	CareLink	Home monitoring
Suspicion of worsening heart failure	OptiVol alert	Mean ventricular rate >80bpm or premature ventricular complex>50/hour
Low biventricular pacing percentage	<90%	<85%
Atrial tachycardia/fibrillation	Atrial tachycardia/fibrillation burden >6hours/day; ventricular rate >100 bpm	Atrial fibrillation episode duration ≥6 hours with concurrent ventricular rate >120 bpm
Nonsustained ventricular tachycardia	Yes	No
Ventricular tachycardia detection	Yes	Yes
Number of shocks delivered	≥1	≥1
Failure of delivered therapy	All therapies exhausted in a zone	Ineffective maximum energy shock
Lead impedance out of range	Yes	Yes
Battery depletion	Recommended replacement time or end of service	Recommended replacement time or end of service
